# Anticancer and Apoptotic Activity in Cervical Adenocarcinoma HeLa Using Crude Extract of *Ganoderma applanatum*

**DOI:** 10.3390/cimb44030067

**Published:** 2022-02-22

**Authors:** Anley Teferra Kiddane, Min-Jae Kang, Truc Cong Ho, Adane Tilahun Getachew, Maheshkumar Prakash Patil, Byung-Soo Chun, Gun-Do Kim

**Affiliations:** 1 Laboratory of Cell Signaling, Department of Microbiology, College of Natural Science, Pukyong National University, 45 Yongso-ro, Nam-Gu, Busan 48513, Korea; alulatef@gmail.com (A.T.K.); minjae@pukyong.ac.kr (M.-J.K.); 2 Department of Food Science and Technology, Pukyong National University, 45 Yongso-ro, Nam-Gu, Busan 48513, Korea; hocongtruc@mku.edu.vn (T.C.H.); bschun@pknu.ac.kr (B.-S.C.); 3 PL Micromed Co., Ltd., 15-5, Yangju 3-gil, Yangsan-si 50620, Gyeongsangnam-do, Korea; 4 National Food Institute (DTU Food), Technical University of Denmark, Kemitorvet, 2800 Kgs. Lyngby, Denmark; atige@food.dtu.dk; 5 Industry-University Cooperation Foundation, Pukyong National University, 45 Yongso-ro, Nam-Gu, Busan 48513, Korea; mahesh@pukyong.ac.kr

**Keywords:** adenocarcinoma HeLa, anticancer, apoptosis, *Ganoderma applanatum*

## Abstract

Cancer is currently one of the foremost health challenges and a leading cause of death worldwide. Cervical cancer is caused by cofactors, including oral contraceptive use, smoking, multiparity, and HIV infection. One of the major and considerable etiologies is the persistent infection of the oncogenic human papilloma virus. *G. applanatum* is a valuable medicinal mushroom that has been widely used as a folk medicine for the treatment and prevention of various diseases. In this study, we obtained crude extract from *G. applanatum* mushroom with a subcritical water extraction method; cell viability assay was carried out and the crude extract showed an antiproliferative effect in HeLa cells with IC_50_ of 1.55 ± 0.01 mg/mL; however, it did not show any sign of toxicity in HaCaT. Protein expression was detected by Western blot, stability of IκBα and downregulation of NFκB, IKKα, IKKβ, p-NFκB-65(Ser 536) and p-IKKα/β(Ser 176/180), suggesting loss of survival in a dose-dependent manner. RT-qPCR revealed RNA/mRNA expression; fold changes of gene expression in Apaf-1, caspase-3, cytochrome-c, caspase-9, Bax and Bak were increased, which implies apoptosis, and NFκB was decreased in a dose-dependent manner. DNA fragmentation was seen in the treatment groups as compared to the control group using gel electrophoresis. Identification and quantification of compounds were carried out by GC–MS and HPLC, respectively; 2(5*H*)furanone with IC_50_ of 1.99 ± 0.01 μg/mL could be the responsible anticancer compound. In conclusion, these findings suggest the potential use of the crude extract of *G. applanatum* as a natural source with anticancer activity against cervical cancer.

## 1. Introduction

Cancer is currently one of the foremost health challenges and a leading cause of death worldwide. According to the World Health Organization (WHO), cancer was responsible for approximately 9.6 million deaths in 2018. Environment (physical carcinogen), certain viral infections (biological carcinogen), and diets (chemical carcinogen) are the main etiologies in the occurrence of cancer [1]. Globally, cervical cancer is one of the most common cancers in women [2]. Cervical cancer is caused by cofactors, including oral contraceptive use, smoking, multiparity, and HIV infection. One of the major and considerable causes is the persistent infection of oncogenic human papilloma virus [3].

Cervical cancer is characterized as an invasive [4] and primary life-threatening cancer disease among women. Cervical cancer arises from pre-existing squamous dysplasia in about 80% of cases and cervical adenocarcinoma is responsible for 20% of invasive cancers. In relation to squamous carcinoma, the occurrence of adenocarcinoma is increasing in more developed countries [5]. Epidemiologic risk factors associated with cervical cancer are a history of smoking, parity, oral contraceptive use, early age of the onset of coitus, a larger number of sexual partners, history of sexually transmitted disease, and chronic immunosuppression [6]. Females infected with HIV/AIDS are at high risk of HPV infection and consequently cervical cancer at an early age (13–18 years) [5]. HIV-positive women with cervical cancer are diagnosed at an earlier age (15–49 years) compared to healthy women [5]. Chronic infection of HPV is the most and main factor in the incidence and progress of invasive cervical cancer in the population [6]. Vaccination to counter HPV is expected to intercept HPV infection for which the vaccine is intended and designed, therefore it prevents cervical cancer caused by certain HPV [6]. Most cases of genital cancer are caused by the HPV, with HPV DNA confirmed in nearly 95% of malignant cervical lesions [7]. HPV serotypes 16 and 18 are reported to account for approximately 70% of cases, with the most common serotypes of HPV in women with cervical cancer, in descending order of frequency, being 16, 18, 45, 31, 33, 52, 58, and 35 [7]. E6 and E7 are the key role players of HPV genes in encoding proteins that are prominent for replication of HPV virus inside the cervical cell [8]. These HPV cancer-causing proteins, E6 and E7, inactivate p53 and pRb tumor suppressor proteins, respectively [9]. Ubiquitin degradation of p53 and inactivation of pRb distort the basic cell cycle checkpoint in HPV 16 or 18 infected cells [8]. Hence, HPV genetic material (DNA) has been diagnosed in cervical adenocarcinomas [5]. Avoidance of HPV infection is able to achieve primary prevention of cervical cancer; however, the effectiveness and reliability of HPV vaccination is much more advanced [5]. Pap smear is the original cervical screening test, and also HPV-based screening is more effective in detecting early cervical cancer; both are helpful in providing prominent prevention versus cervical cancers [5]. The successful implementation of a cervical cancer screening program for cervical cancer detection and treatment has resulted in relatively significant mitigation in cervical cancer prevalence and mortality [10].

Conventional cancer treatments, including surgery, radiation, and chemotherapies, have unpleasant side effects; hence, interests are growing in seeking natural ingredients with fewer side effects, less toxicity, and high efficacy for cancer prevention and treatment [11]. Mushrooms act as both functional food and medicine, achieve various bioactivities, and make a great contribution to health-promoting products. They contain attractive nutritional composition and several classes of bioactive compounds, such as alkaloids, steroids, polyphenols, polysaccharides [12,13], fatty acids, micronutrients, minerals, and vitamins [14,15]. Mushrooms are found to be potent in curing diseases, mainly by the mechanism of regulating cell surface receptors. Antioxidant, anticancer, anti-inflammatory, and immunomodulatory possessions of mushrooms are major tasks among diverse biological activities [13]. *G. applanatum* is one of the wild mushroom species which attaches and grows on the wood; it is a valuable medicinal mushroom that has been extensively utilized as a folk medicine for the treatment and prevention of various diseases [16].

So far, numerous scientific investigations have been underway regarding mushrooms, including *G. applanatum*, though research regarding the association between *G. applanatum* and its anticancer activity on cervical cancer is negligible. Therefore, the objective of this study is to investigate the in vitro apoptosis pathway determination and anticancer activity of the crude extract of *G. applanatum* in human adenocarcinoma HeLa cells.

## 2. Materials and Methods

### 2.1. Crude Extraction

The dried mushrooms were cut manually into smaller pieces by using a knife and scissors, which helps to increase the surface area of the sample to allow it to dry as fast as possible, and also the smaller size is easy to grind in the machine. The mushrooms were then put into a convection oven for 72 h at 40 °C until dry, as required. The dried sample was ground and turned to powder; 20 g of sample powder was weighed and mixed with 600 mL distilled water. The extraction was carried out by using the subcritical water extraction method [17]. The extraction condition of the machine set-up was adjusted at 140 °C temperature, 30 bar pressure, and 200 RPM stirring speed for 50 min, then filtered twice by using 110 mm diameter filter size (glass microfiber filters, GF/A, 100 circles, Cat. No. 1820-110) to make it purer, then 300 mL filtered sample was mixed with 900 mL of 99.99% ethanol and kept in −80 °C deep freezer for an hour. Then, it was put into a freeze drier machine (Hypercool (HC4110), Gyrozen Co. Ltd., Daejeon, Korea) at −91 °C for days. Finally, the crude extract (GAMCE) was obtained.

### 2.2. Cell Culture

HaCaT (AddexBio, San Diego, CA, USA) and HeLa cell lines (ATCC, Manassas, VA, USA) were used for this study and kept in a humidified atmosphere with 5% CO_2_ at 37 °C by using DMEM (Mediatech, Manassas, VA, USA) for HaCaT cell, MEM (Mediatech, Manassas, VA, USA) for HeLa cells culturing. Then, when cells became 80% confluent, they were sub-cultured until they reached passage-3 and were ready for the experiment. DMEM and MEM were supplemented with 10% heat-inactivated fetal bovine serum (Mediatech, Woodland, CA, USA) and 1.1% antibiotic antimycotic (Mediatech, Manassas, VA, USA).

### 2.3. Solution Preparation

100 mg/mL concentration of GAMCE stock was prepared by diluting 15 mg of GAMCE in 150 µL of 25% DMSO. Stock were diluted in each specific media and prepared in different concentrations; (1.4, 1.5, 1.6 and 1.7 mg/mL) for HeLa and HaCaT cells treatment.

### 2.4. Cell Viability Assay

To determine the cytotoxicity of the GAMCE, the control, and experimental group cells were seeded in a 96-well plate with a density of 1 × 10^4^ cells in a single well and a blank well with 100 µL media without cells. They were incubated at 37 °C 5% CO_2_ condition for 24 h. After 24 h, HeLa and HaCaT experimental groups were treated with 1.4, 1.5, 1.6, and 1.7 mg/mL concentrations of the GAMCE and incubated for 24 h. After 24 h, media was changed and replaced with fresh media containing EZ-Cytox solution (10 µL) in the blank, control, and experimental group wells, also protected from light and incubated for 2 h at 37 °C 5% CO_2_. The cell viability was calculated with the ELISA plate reader at 460 nm (Varioskan Lux, Vantaa, Finland). Cell viability was calculated as [(experimental-blank) ÷ (control-blank)] × 100. This experiment was carried out in triplicate.

### 2.5. Protein Extraction and Westernblot

A total of 25 mL of MEM constituted HeLa cells suspension (adjusted: 1 × 10^5^ cells/mL) was prepared, then 5 mL was inoculated on each 100 mm cell culture dish incubated at 37 °C with 5% CO_2_ for 24 h. The media were then changed, and the control group (no treatment) and the experiment group were treated with 1.4, 1.5, 1.6, and 1.7 mg/mL of GAMCE and incubated at 37 °C with 5% CO_2_ for 12 h. Subsequently, the media was removed, HeLa cells were washed, scraped and collected with cold PBS, transferred into 15 mL conical tubes. Following by centrifugation with 1400 RPM for 5 min. After centrifugation cells are lysed by the addition of 35 µL cell lysis buffer (iNtRON BIOTECHNOLOGY, Cat. No. 17081, Gyeonggi-do, Korea). Incubated for 10 min on ice, lysates were collected and clarified by centrifugation at 14,000 rpm for 20 min at 4 °C. Protein quantification was carried out by using Albumin bovine 2 mg standard (Lot# SA242714A, Thermo Scientific, Rockford, IL, USA) and Bradford reagent for ELISA microplate reader at 595 nm. Aliquots of whole-cell lysates were formed on 12% SDS-PAGE and bound to the nitrocellulose membrane. The membranes were blocked with 5% skim milk in PBST (PBS buffer and 0.5% Tween-20). After blocking non-specific sites, the membranes were probed with primary antibodies (Cell Signaling Technology, Danvers, MA, USA) and then washed in PBST three times, followed by incubation for 1 h with anti-rabbit IgG or anti-mouse IgG as secondary antibodies (Cell Signaling Technology, Danvers, MA, USA). The blots were then washed in PBST and visualized by an enhanced chemiluminescent detection solution (Abfrontier, Lot.QJN28, Seoul, Korea) and imaging system (Thermo Fisher Scientific, iBrightCL1000, Waltham, MA, USA). Finally, Band density of the blotted image was measured by using Adobe photoshop CS6.

### 2.6. RNA Extraction

15 mL of MEM constituted HeLa cells suspension (adjusted: 1 × 10^5^ cells/mL) was prepared, then 5 mL was inoculated on each 100mm cell culture dish and incubated at 37 °C with 5% CO_2_ for 24 h. The media were then changed, and the control group (no treatment) and the experiment group were treated with 1.5 and 1.6 mg/mL of GAMCE and incubated at 37 °C with 5% CO_2_ for 12 h. Next, the media was removed and then 1 mL cold PBS was added in each dish, the control with no treatment and GAMCE (1.5 and 1.6 mg/mL) treated cells were scraped and collected into separated 1.5 mL-eppendorf tubes. Centrifuged at 8000 RPM for 10 min at 4 °C. RNA was extracted using (RNeasy^®^ Mini Kit (50); QIAGEN, Hilden, Germany) with protocol.

### 2.7. Reverse Transcriptase-PCR

Extracted RNA was transcribed to cDNA by adding the proper amount of RNA and RNase-free water in a SuPrimeScript RT Premix (2X with oligo dT; GeNet Bio, Global Gene Network, Daejeon, Korea), and kept in PCR machine (SampliAmp Thermal cycler, Singapore), incubated at (50 °C for 60 min, then 70 °C for 10 min) 1-cycle. Finally kept at 4 °C.

### 2.8. Real Time-qPCR

RT-qPCR was carried out to observe the relative fold change of genes expression in HeLa cells. This was conducted by mixing 7 μL distilled water, primer (Table 1) (1 μL forward and 1 μL reverse) (Bioneer, Daejeon, Korea), 1 μL cDNA, 10 μL qPCR master-mix (Bioneer, Daejeon, Korea) in each qPCR tubes (Bioneer, Daejeon, Korea). Spun down the tubes and run in triplicate using RT-qPCR machine (Exicycler^TM^ 96; Bioneer, Korea) with a qPCR condition at (95 °C for 5 min) 1-cycle, then at (95 °C for 20 s; 60 °C for 40 s; 72 °C for 30 s) 45-cycles. The relative quantification result was determined by 2^−∆∆C^_T_ method [18].

### 2.9. DNA Fragmentation

15 mL of MEM constituted HeLa cells suspension (adjusted: 1 × 10^5^ cells/mL) was prepared, then 5 mL was inoculated on each 100mm cell culture dish and incubated at 37 °C with 5% CO_2_ for 24 h. After 24 h, the media were changed, and the control and experimental groups treated with GAMCE concentrations of 1500 and 1600 µg/mL incubated for 24 h. Next, the media were removed and then 1 mL cold PBS was added in each dish, cells were scraped and collected into separated 1.5 mL-eppendorf tubes. Genomic DNA was extracted from the control and experiment groups using a kit (AccuPrep^®^ Genomic DNA Extraction Kit; Bioneer, Gyeonggi-do, Korea) with protocol. Finally, DNA fragmentation was checked using 1.5% agarose gel electrophoresis with 100 V run for 30 min followed by an imaging system (Thermo Fisher Scientific, iBrightCL1000, Waltham, MA, USA).

### 2.10. GC–MS

Identification of compounds was carried out by using gas chromatography–mass spectrometry (GC–MS) (Model Name: GCMS QP-2010Ultra, Shimadzu, Kyoto, Japan) and Column (DB-5MS Ultra (30 × 0.25 × 0.25)). The sample was dissolved in water, diluted with methanol, and the precipitated portion was centrifuged and analyzed as a supernatant.

### 2.11. HPLC

Slightly modified quantitative HPLC analysis was performed in accordance with the protocol discussed by Pérez et al. (1996). HPLC fit out with Luna 5μ C18(2) 100A New Column 250 × 4.6 mm was operated. UV detector with a wavelength of 280 nm and an injection volume of 20 μL were set. The mobile phase used to separate furanone consists of two eluents: 0.2 M sodium acetate/acetic acid, pH 4 (solvent A), and methanol (solvent B). Chromatographic conditions were 0–11 min, isocratic 13% B; 11–26 min, gradient 13–23% B; 26–30 min, isocratic 23% B; and 30–33 min, gradient 23–80% B (cleaning process) [19].

### 2.12. Statistical Analysis

All data are presented and shown as Mean ± SE. Statistical analysis was conducted with ANOVA and *t*-test using Microsoft Excel where the mean value of control group was individually compared with the mean value of every treated group. *p*-value was used as statistical significant.

## 3. Results

**Figure 1:**
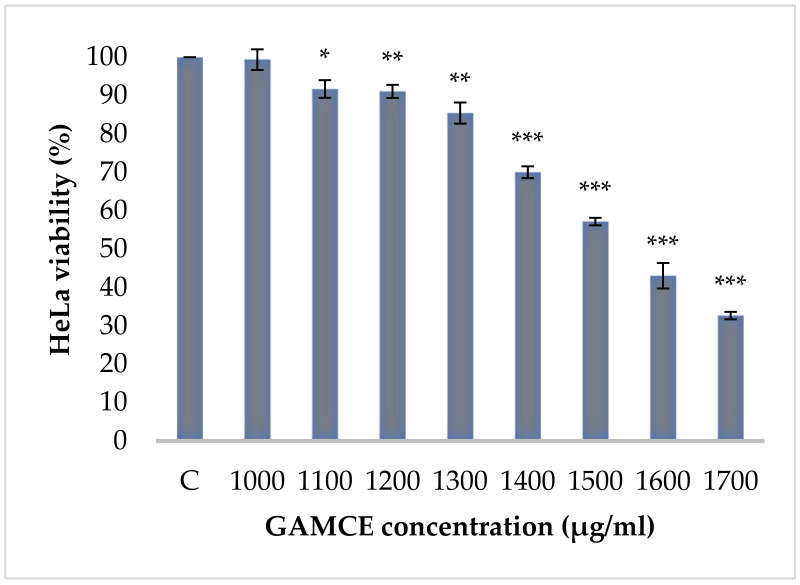
HeLa cells, control group (C = 100% viability) was not treated at all; experimental groups were treated with different concentrations (1000, 1100, 1200, 1300, 1400, 1500, 1600, and 1700 μg/mL) of GAMCE for 24 h. The test result of IC_50_ of GAMCE was 1.55 ± 0.01 mg/mL. The data are shown as Mean ± SE. Mean significant difference (* *p* < 0.05, ** *p* < 0.01, *** *p* < 0.005) compared to control.

**Figure 2:**
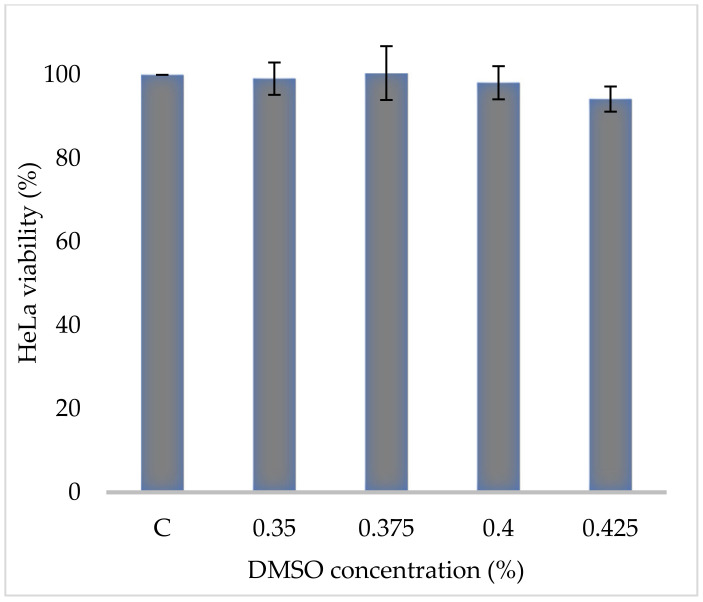
HeLa cells, control group (C = 100% viability) was not treated at all; experimental groups were treated with different concentrations (0.35, 0.375, 0.4, and 0.425 %) of DMSO for 24 h. The result did not show any significant difference. The data are shown as Mean ± SE. Mean significant difference compared to control.

**Figure 3:**
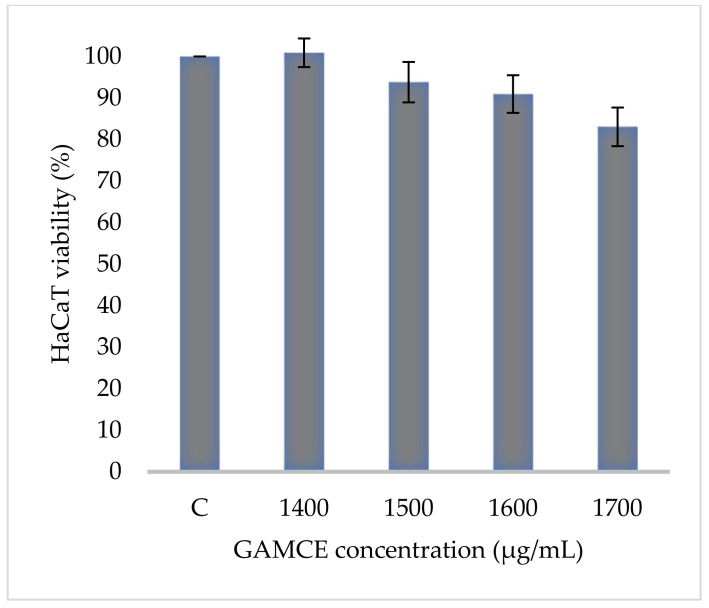
HaCaT cells, control group (C = 100% viability) was not treated at all; experimental groups were treated with different concentrations (1400, 1500, 1600, and 1700 μg/mL) of GAMCE for 24 h. The result did not show significant difference. The data are shown as Mean ± SE. Mean significant difference compared to control.

**Figure 4:**
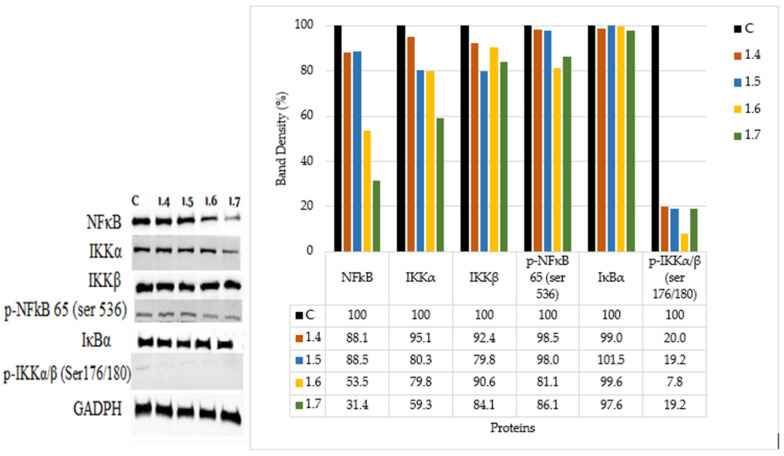
Western blot result of expressed protein signals by HeLa cells control group (with no treatment) and experimental groups treated with different concentrations (1.4, 1.5, 1.6, and 1.7 mg/mL) of GAMCE for 12 h. Band density of the blotted image was measured by using Adobe photoshop CS6.

**Figure 5:**
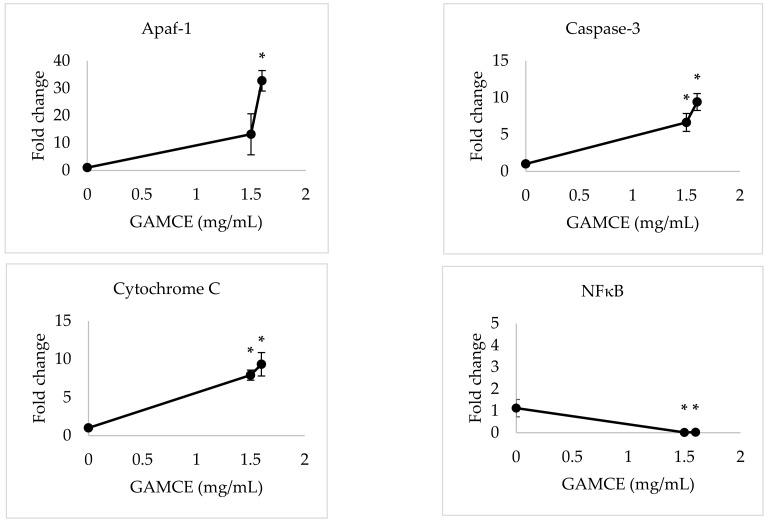

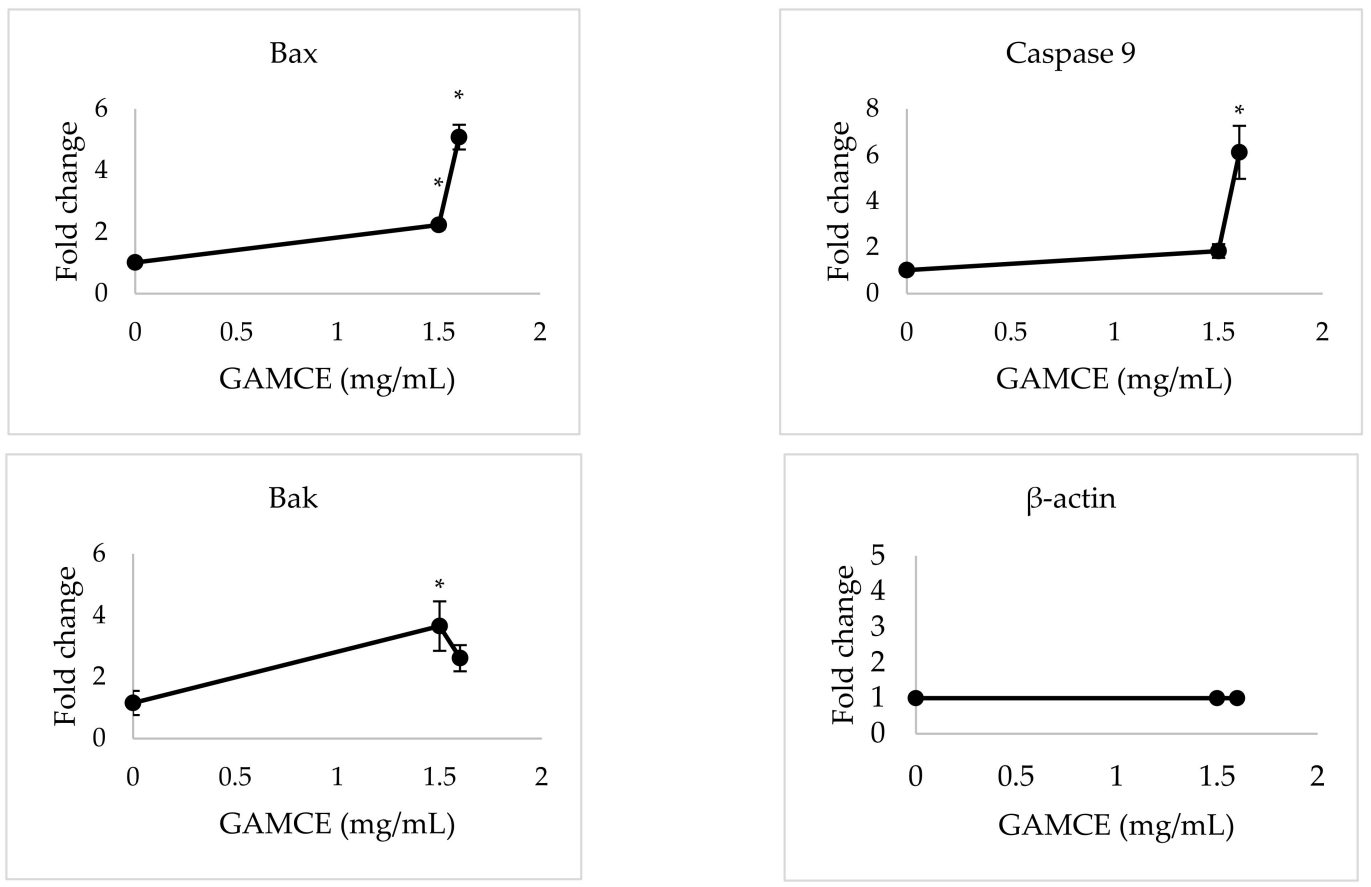
RT-qPCR results of relative gene expressions of GAMCE (1.5 and 1.6 mg/mL) treated HeLa cell lines compared with the control group (0 mg/mL) with no treatment, of 12 h incubation. The data are shown as Mean ± SE. Mean significant difference (* *p* < 0.05) compared to control.

**Figure 6:**
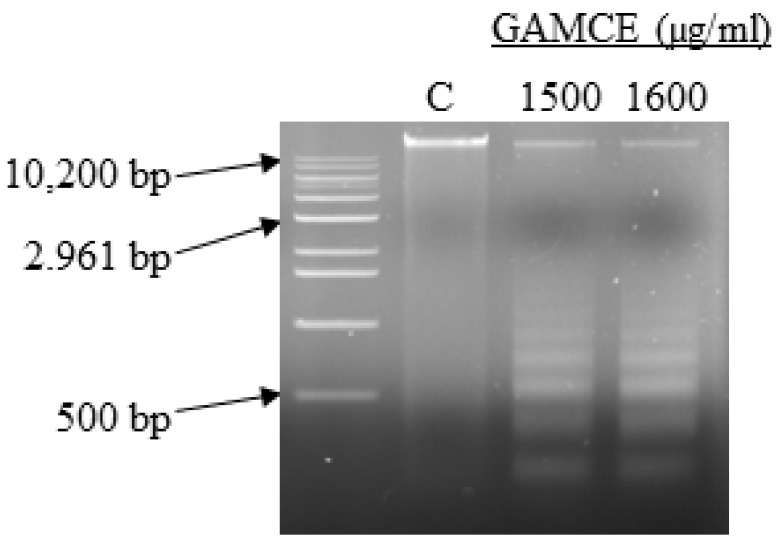
DNA fragmentation of GAMCE (1500 and 1600 µg/mL) treated HeLa cells for 24 h as compared to the control, HeLa genomic DNA was extracted and separated by 1.5% agarose gel electrophoresis with 100 V run for 30 min.

**Figure 7:**
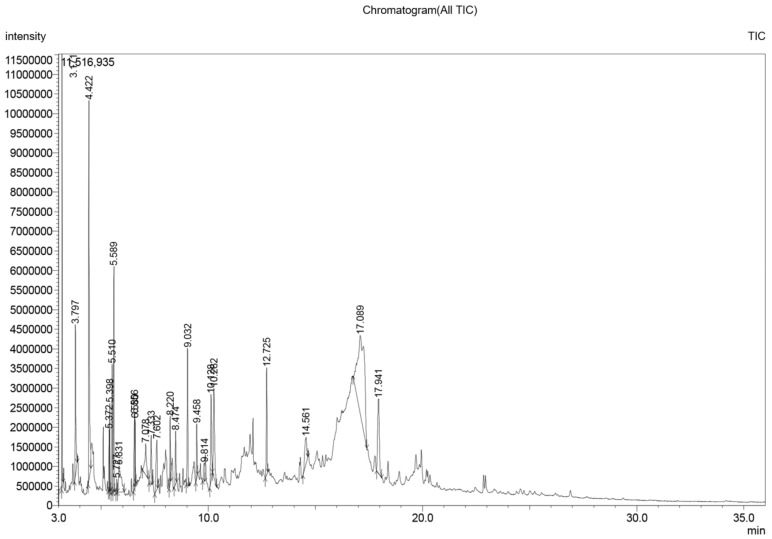
GC–MS chromatogram of crude extract of *G. applanatum*.

Crude extraction was carried out by subcritical water extraction method and 820 mg of freeze-dried GAMCE was obtained and collected from 20 g of sample powder input. Cell viability was carried out on HeLa and HaCaT cell lines in vitro; the results obtained with IC_50_ of GAMCE for HeLa was 1.55 ± 0.01 mg/mL (Figure 1) when compared with the control group and DMSO didn’t show significant toxicity on HeLa (Figure 2) compared to the control group. Additionally, GAMCE did not show significant toxicity on HaCaT cells (Figure 3). Western blot (Figure 4) was performed to determine the signaling proteins’ expression change responsible for the alteration in proliferation after treatment compared with the control group. Proliferation enhancer proteins, NFκB, IKKα, IKKβ, pIKKα/β (Ser176/180), and p-NFκB-65(Ser 536), were detected and their expression gradually downregulated related to the dose-dependent manner. Furthermore, the level of expression of apoptotic genes, which are responsible for cell death, and a transcriptional factor (NFκB), were investigated using RT-qPCR, and the test exhibited the fold change of gene expression (Figure 5) in the control, 1.5 and 1.6 mg/mL treated HeLa cells; caspase-3 was 1.01 ± 0.11, 6.64 ± 1.21, and 9.41 ± 1.14; Apaf-1 was 1.02 ± 0.14, 13.18 ± 7.49, and 32.72 ± 3.75; cytochrome-C was 1.00 ± 0.06, 7.91 ± 0.66, and 9.34 ± 1.53; NFκB was 1.13 ± 0.39, 0.01 ± 0.00, and 0.02 ± 0.00; Bax was 1.01 ± 0.10, 2.23 ± 0.14, and 5.08 ± 0.40; caspase-9 was 1.01 ± 0.1, 1.84 ± 0.3, and 6.12 ± 1.15; Bak was 1.15 ± 0.39, 3.65 ± 0.80, and 2.61 ± 0.43, respectively. One of the hallmarks of apoptotic cell death is the breakdown of nuclear DNA into nucleosomal fragments. In this experiment, higher DNA fragmentation was seen in the treatment groups as compared to the control group using gel electrophoresis (Figure 6). GC–MS analysis identified various compounds presented in the extract (Figure 7 and Table 2) for cross-checking with other studies and estimating certain molecules, and then providing a recommendation for further research. Finally, quantitative HPLC analysis showed 1284.29 ± 12.4 μg of 2(5*H*)furanone in the 1 g of GAMCE; accordingly, the compound 2(5*H*)furanone with IC_50_ of 1.99 ± 0.01 μg/mL available in the crude extract with IC_50_ of 1.55 ± 0.01 mg/mL, could be responsible for the antiproliferative activity.

## 4. Discussion

The aim of this research is to help and serve humanity in discovering suitable and effective therapeutic sources against cervical cancer. Crude extracts of natural sources are able to demonstrate numerous biological activities, including anticancer activity, and research reports are increasing. Specifically, this study focuses on treating and curing cervical cancer by using crude extract of *G. applanatum* mushroom as a natural ingredient. The crude extract derived from the *G. applanatum* own anticancer activity effect in HeLa cells was observed by killing 50% HeLa cells at a 1.55 ± 0.01 mg/mL concentration level in vitro. This result showed the anti-cervical cancer potency of the crude extract of *G. applanatum* in HeLa cells.

Apoptosis is a special type of cell death program that can trigger either the extrinsic or intrinsic apoptotic pathway [20]. NFκB is a heterodimer protein complex that controls DNA transcription and innate immunity regulator [21] and inflammatory cytokines [22] for cell survival. Increasing evidence suggests that NFκB activation is associated with resistance to apoptosis and carcinogenesis due to its important effects on cell differentiation and proliferation in malignancies [22]. In most cells, NFκB dimers are present in the cytoplasm as a dormant configuration in a bind with any large number of IκB blockers (IκB α, β, ε, γ, p105, and p100) [23]. IκB is rapidly phosphorylated, ubiquitinated, and degraded by the proteasome, and the released NFκB dimer is then transferred to the nucleus, where it can regulate specific gene expression [24]. Phosphorylation and degradation of IκB have received considerable attention as key steps in regulating NFκB complexes [25]. Several molecules maintain high levels of IκB protein in the cytoplasm in order to inhibit NFκB and thus preventing translocation of NFκB to the nucleus [26] following the treatment. Among these molecules, some promote the synthesis of IκBα, some inhibit IκBα ubiquitination, while others block IκBα degradation [23]. Accordingly, ubiquitin-proteasome inhibitors of any step inhibit NFκB activation by stabilizing IκB [27]. IKK protein is phosphorylated at two serine residues (Ser176/180 of IKKα or Ser 177/181 of IKKβ) and subsequently induces the phosphorylation of IκBα at Ser 32/36 [28]. Significantly inhibiting the phosphorylation of p-IKKα (Ser176/180) and p-IκBα (Ser32) then leads to a decrease in nuclear levels of NFκB p65 [28]. Various anticancer drugs have shown anti-NFκB activity, which may be related to their anti-tumor effects, possibly by reducing NFκBs proliferative and anti-apoptotic activity [29]. We investigated the various signaling pathways and discovered the change in the aforementioned proteins. The conclusion drawn in this study is that the downregulation of p-IKKα/β (Ser176/180), the stabilized expression of IκBα, and the downregulation of NFκB were related to dose-dependent manner of GAMCE treatment. This manipulation of proteins and signal alteration, which is the cause of HeLa cell death in the experiment, might be caused by the crude extract’s action. The findings showed that GAMCE possesses an apoptosis-inducing effect through altering the above-mentioned signaling pathways and proteins, as well as preventing the translocation of NFκB into the nucleus, which is a key player in cancer cell proliferation and survival. The Western blot experiment has proven this to be true.

The interaction between anti-apoptotic and pro-apoptotic members can control the integrity of mitochondria and take a crucial part in regulating the release of mitochondrial cytochrome-c [30]. Additionally, various conditions of intracellular stress induce apoptotic cell death by stimulating the release of cytochrome-c from mitochondria to the cytoplasm. Bcl-2 family proteins are important participants in the cell apoptosis. Bak and Bax are considered to be homo-oligomerized in forming pores on the outer mitochondrial membrane [31], thus cause cytochrome-c deployment into cytosol [32]. In the intrinsic apoptotic pathway, cytochrome-c deployed from mitochondria associates with Apaf-1 protein and oligomerize to initiate large complex apoptosome-mediated activation of caspase-9 and then caspase-3 [32]. Procaspase-9 is the initiator caspase in the mitochondrial pathway, which is mobilized and activated by the apoptosome that triggers the downstream caspase-3 [20]. Then, active caspase-9 or caspase-8 activates caspase-3 to perform apoptosis [32]. Caspase-3 protein expressions in drug-treated cells are expected to change if there is declined membrane potential. Hence, caspase-3 expressions are considered as revealing indicators of apoptosis [33]. Increased caspase-3 activation is coordinated with changes in the family of Bcl-2, including the upregulated Bax and Bak, and downregulated Bcl-2 protein expression [30]. The activation of a critical transcription factor NFκB, which stimulates the expression of genes producing cell survival proteins, is usually the mechanism by which cells survive. Caspases also disrupt the generation of survival signals by inactivating the NFκB pathway [34]. Inhibition of the NFκB pathway might be a useful target to sensitize tumor cells to TNFα-induced-apoptosis [33]. These apoptotic proteins are made by specific genes, so in this investigation we designed and performed a way to detect the response of these manipulative genes to the treatment. The expression of apoptotic genes caspase-3, Apaf-1, cytochrome-c, caspase-9, Bax and Bak upregulation and the reduction of NFκB gene was observed after treating HeLa cells with GAMCE. These results indicate that there was a phenomenon of apoptosis inside the cells related to the treatment because these apoptotic genes have their own natural potency and ability to synthesize apoptotic proteins and induce impulse for apoptotic cell death. This fundamental principle leads us to the conclusion that GAMCE therapy on HeLa initiated intrinsic signaling for the activation of apoptosis.

Apoptosis in HeLa is characterized biochemically by the cleavage of chromosomal DNA into oligonucleosomal-sized pieces after the treatment with the crude extract. Apoptotic stimuli can cause cells to die without causing DNA degradation, while DNA fragmentation might hasten the process. Later phases of the apoptotic process in HeLa resulted in DNA fragmentation. Furthermore, the caspase family predominantly mediates apoptotic signal pathways, which finally result in DNA fragmentation. However, both caspase-dependent and caspase-independent apoptotic DNA fragmentation may have the possibility of occurring in HeLa after the treatment. The DNA ladder gel electrophoresis method was performed in this investigation (Figure 6), and it demonstrated the presence of considerable DNA fragmentation in the GAMCE treated groups as compared to the control group.

**Figure 8:**
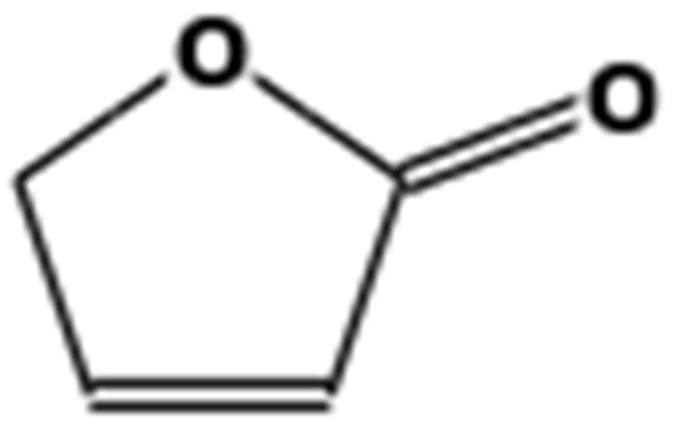
Chemical structure of 2(5*H*)-furanone.

Furanone is a five-membered heteroaromatic ring containing oxygen atoms, which is of great significance in pharmaceuticals. The presence of this bio-active compound in natural and artificial origin has played an essential role in the design and development of novel drugs. These days, the synthesis of furanone derivatives and the exploration of their medicinal effects have been some of the main concerns of researchers [35]. Furanone is divided into three categories: 2(3*H*)-furanone, 2(5*H*)-furanone, and 3(2*H*)-furanone [36]. 2(5*H*)-furanone (Figure 8) units are widely present in many natural products as structural components and skeletons [37]. There have been various studies showing that 2(5*H*)-furanone is a commonly used conduit for many drugs that have a variety of bio-activities, such as antifungal, antibacterial, and anti-inflammatory. Many 2(5*H*)-furanone derivatives have also been reported as antitumor agents in nature [38,39]. According to the study [37], 2(5*H*)-furanone derivative has shown cytotoxicity effect on MCF-7 breast cancer cells, and cytotoxicity against A549 cells has been observed [40]. Chiral dithiocarbamates with group 2(5*H*)-furanone-piperazine shows effective cytotoxicity against HeLa and SMMC-7721 human hepatocellular carcinoma cells [41]. In vitro assays of a related molecule 5-(bromomethyl)furan-2(5*H*) showed to promote apoptosis and cell cycle arrest in HepG2 (liver) and A549 (lung) human neoplastic cells, mediated by mitochondrial extrinsic, or death receptor pathways [39]. 5-Arylene-2(5*H*)-furanone derivatives show effective cytotoxicity on A549 and SK-OV-3 cell lines [42]. Bis-2(5*H*)-furanone derivatives show acceptable antiproliferative activity against cancer cell lines, including C6 (rat glioma cell), EC-1 (human esophageal carcinoma cell), MDA-MB-231 (estrogen receptor-negative human breast cell), MCF-7 (estrogen receptor-positive human breast cell), HepG2 (human hepatoma cell), and CNE-1 (nasopharyngeal carcinoma cell) [43]. The resulting dihydrofurofuranone shows significant anti-tumor activity on breast cancer cell lines, and the molecule can activate the intrinsic apoptotic mechanism [44]. These results and scientific shreds of evidence indicate promising potential in implementation and value of 2(5*H*)furanone in cancer chemotherapy. The anticancer action of *G. applanatum* may be attributed to the 2(5*H*)furanone ingredient in the crude extract; however, further research is needed to make the estimation more reliable and tangible.

## 5. Conclusions

In this study, there are apparent and objective changes observed on HeLa cells’ viability, gene expression, protein expression, and DNA fragmentation test results after treating the cells with the crude extract of *G. applanatum* mushroom. These significant outcomes lead us to conclude that the crude extract of *G. applanatum* mushroom has natural therapeutic potency in the treatment for cervical cancer: by launching intrinsic apoptotic suicidal signals, by suppressing DNA survival and proliferation transcription factors, by causing DNA fragmentation, and by ceasing the growth and the metastatic prospect of the cervical cancer cells.

Rather than this preliminary report, additional research is recommended to observe the application, route, dose, pharmacodynamics, pharmacokinetics, and other properties of the crude extract and specific isolated compounds, as well as to implement anticancer therapy products as a drug or supplement in the commercial field.

## Figures and Tables

**Table 1:** List of designed primers used for RT-qPCR.

Genes	Product Size	Sequences
NF-kB	104 bp	Forward 5′-GGGAGATGAACCCTGCCAAA-3′
		Reverse 5′-AAAGTGGTGTTCCCAGGCAA-3′
Caspase-3	102 bp	Forward 5′-CTGTGAACCCTGCATTTGGC-3′
		Reverse 5′-ACTTCGGAAGCTGAACCTGG-3′
Cytochrome-c	104 bp	Forward 5′-TGGCTTAATGTGTTCGCCCT-3′
		Reverse 5′-AAGCCCAAGCAAAGAGGGAA-3′
Apaf-1	101 bp	Forward 5′-TGGGTGACTGACCTTTGCTTT-3′
		Reverse 5′-GTCTGTGAGGATTCCCCAGTG-3′
Bax	102 bp	Forward 5′-ACGAGGGTGATAGGTGGTACA-3′
		Reverse 5′-TGTTCTTCCCTTACCCACACG-3′
Caspase-9	105 bp	Forward 5′-GAAGAGACCTGGCCAGAACC-3′
		Reverse 5′-ATTGCACAGCACGTTCACAC-3′
Bak	101 bp	Forward 5′-GGTTTTCCGCAGCTACGTTTT-3′
		Reverse 5′-GTTGCAGAGGTAAGGTGACCA-3′
β-actin	104 bp	Forward 5′-TCTTCCAGCCTTGCTTCCTG-3′
		Reverse 5′-GGTGTACAGGTCTTTGCGGA-3′

**Table 2:** List of compounds identified in the crude extract of *G. applanatum* by using GC–MS.

Peak	Retention Time	Area%	Name
1	3.171	6.77	2-Propanone, 1-hydroxy-(CAS)
2	3.797	3.96	Acetamide (CAS)
3	4.422	10.25	Dimethyl sulfoxide-D(6)
4	5.372	1.43	2(5*H*)-furanone
5	5.398	1.79	2(3*H*)-furanone, dihyfro-(CAS)
6	5.510	3.56	-
7	5.589	5.65	2-Cyclopenten-1-one, 2-hydroxy-
8	5.727	0.27	2(5*H*)-Furanone, 5-methyl- (identity?) (CAS)
9	5.831	2.98	2-Furancarboxaldehyde, 5-methyl- (CAS)
10	6.556	2.40	2-Hydroxy-gamma-butyrolactone
11	6.580	1.18	“5,6–dihydro–pyran–2,5–di–one” (so Pastorova) questiona
12	7.078	2.07	-
13	7.333	1.94	4-Heptanone (CAS)
14	7.602	2.53	2,5-Dimethyl-4-hydroxy-3(2*H*)-furanone
15	8.220	1.66	Anhydro-sugar
16	8.474	1.61	-
17	9.032	4.01	4H-Pyran-4-one, 2,3-dihydro-3, 5-dihydroxy-6-methyl-(CAS)
18	9.458	1.48	2-Butenethioic acid, 3-(ethylthio)-, S-(1-methylethyl) ester
19	9.814	0.95	1,2-Benzenediol (CAS)
20	10.128	3.48	1,4:3,6-Dianhydro-.alpha.-d-glucopyranose
21	10.262	4.96	5-Hydroxymethylfurfural
22	12.725	3.37	-
23	14.561	2.36	-
24	17.089	23.94	DL-Arabinitol
25	17.941	5.41	-

## Data Availability

Not applicable.

## Abbreviations

Apaf-1	Apoptotic protease activating factor-1
DMEM	Dulbecco’s Modified Eagle’s Medium
GAMCE	Crude extract of *Ganoderma applanatum*
GC-MS	Gas chromatography-mass spectrometry
HaCaT	Human keratinocyte
HeLa	Adenocarcinoma of the cervix
HPV	Human papilloma virus
IC_50_	50% inhibitory concentration
MEM	Minimum Essential Medium
PBS	Phosphate buffered saline
WHO	World Health Organization
